# An Unusual Case of Meyerson Phenomenon Around Infantile Hemangioma

**DOI:** 10.5826/dpc.1103a39

**Published:** 2021-07-08

**Authors:** Giulia Veronesi, Miriam Leuzzi, Annalucia Virdi, Carlotta Gurioli, Iria Neri

**Affiliations:** Dermatology Department of Experimental, Diagnostic, and Specialty Medicine, University of Bologna, Bologna, Italy

**Keywords:** Meyerson phenomenon, hemangioma, eczema, dermoscopy

## Introduction

Meyerson phenomenon (MP) is characterized by an eczematous halo around a pre-existing skin lesion. This phenomenon has frequently been described around melanocytic lesions or non-melanocytic skin neoplasms. Only few cases related to vascular lesions have been reported in the literature.

## Case Presentation

**Figure 1:**
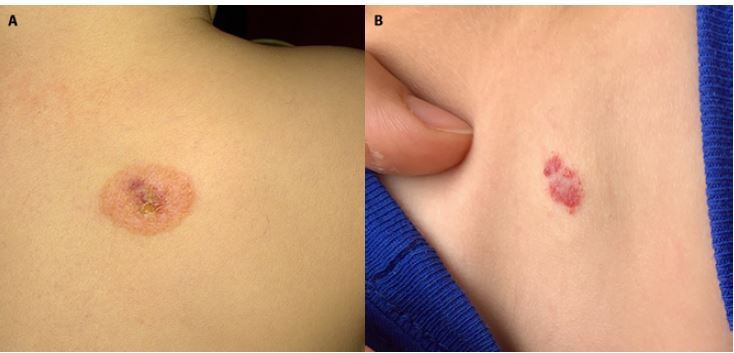
Physical evaluation of infantile hemangioma with Meyerson phenomenon. (A) Purple-red plaque of 3.5 × 2.5 cm in diameter, surrounded by yellowish crusts and an erythematous scaly halo on the back. (B). Total resolution after 2 weeks with topical steroid therapy

**Figure 2:**
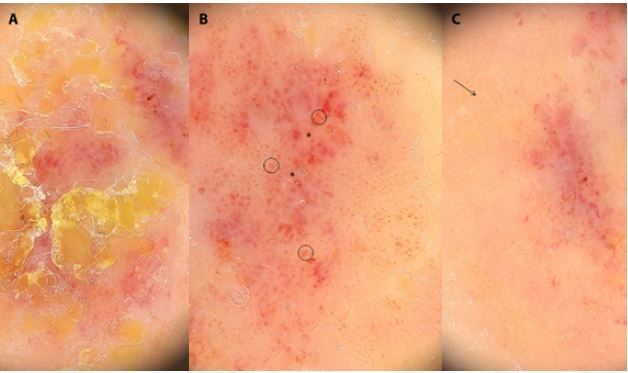
Dermoscopic evaluation of infantile hemangioma with Meyerson phenomenon. (A) Yellow sero-crusts on the lesion. (B) Polymorphous vascular patterns, after crusts dissolution, with red glomerular vessels, circle vessels, comma-like vessels and hairpin vessels (circles) surrounded by whitish septa (*). (C) A rim of erythema with dotted vessels and white-yellow scales on periphery (arrow)

A 10-month-old Caucasian boy was referred to us for a worsening infantile hemangioma with fast clinical changes and itch onset. On physical evaluation, a purple-red plaque surrounded by yellowish crusts and an erythematous scaly halo of 3.5 × 2.5 cm in diameter was observed on the back (Figure 1A). Parents reported that the current eczematous halo had abruptly developed around the vascular lesion 2 months before without no specific trigger. No family and personal history of atopic dermatitis, psoriasis, or allergy were reported. A second superficial infantile hemangioma, with no eczematous modifications, was detected on the patient’s left arm. Dermoscopy revealed yellow sero-crusts (Figure 2A). After their dissolution, a central polymorphous vascular pattern with red glomerular vessels, circle vessels, comma-like vessels, and hairpin vessels surrounded by whitish lobular septa was disclosed (Figure 2B). A rim of erythema with dotted vessels and white-yellow scales characterized the vascular lesion periphery (Figure 2C). The patient was diagnosed with infantile hemangioma (IH) with MP and was prescribed local treatment with fluticasone propionate once a day, with total resolution after 2 weeks (Figure 1B).

## Discussion

In 1971, Meyerson first described an eczematous halo dermatitis surrounding a preexisting melanocytic nevus [1]. The pathogenesis of MP is still unknown. Some authors have suggested an autoimmune reaction against skin melanocytes [1]. CD4 T lymphocytes and intercellular cell adhesion molecule 1 (ICAM-1) were the main suspects in this immune mediated phenomenon [1]. Other Authors have reported that ultraviolet radiations can trigger the inflammatory process [1]. However, the MP is not limited to melanocytic nevi and it can be present in numerous other conditions. Only few reports on MP in vascular lesions have been reported, regarding solitary angiokeratoma or nevus flammeus [2]. To the best of our knowledge, this is the first case related to an infantile hemangioma. In our case, dermoscopy supported the diagnostic process. It showed red lacunae with polymorphous vascular patterns surrounded by whitish septa in the center of the lesion, dotted vessels, and white-yellow scales at the periphery. Over the last years, dermoscopy has increasingly been used in dermatological clinical practice. Morphology of vascular structure and vascular pattern have been described in literature helping the correct recognition of skin tumors, infection, or inflammatory disease [3]. Reddish and oval lacunae surrounded by whitish septa are the typical dermoscopic finding in IH [3]. The perilesional halo of eczema with dotted vessels and white-yellow scales oriented the MP diagnosis. The pathogenesis in vascular cases of MP is again not clear [2]. One hypothesis is that eczematous lesions on nevus flammeus can be the result of a collision with some dermatoses such as atopic dermatitis. However, atopic dermatitis on vascular lesions has a different clinical presentation compared to MP. Another theory suggests that vascular malformation, together with vasodilatation, ectatic capillary vessels or altered endothelial cells, might play a role in the inflammatory process [2]. This occurs with an abnormal production of proinflammatory cytokines and the development of eczema [2]. A similar hypothesis may be supposed also in our case. However, the patient presented 2 superficial IHs and MP was observed only in the back region. This leads us to think that some local triggering events, not studied yet, may have played an important role in the pathogenetic mechanism, and will be evaluated in the future.
